# Percutaneous Abscess Drainage: Basic Techniques and Technical Tips

**DOI:** 10.7759/cureus.95980

**Published:** 2025-11-03

**Authors:** Yasuyuki Onishi, Hironori Shimizu, Shunsuke Sugawara, Hirotsugu Nakai, Yuji Nakamoto

**Affiliations:** 1 Diagnostic Imaging and Nuclear Medicine, Kyoto University, Kyoto, JPN; 2 Diagnostic Radiology, National Cancer Center Hospital, Tokyo, JPN

**Keywords:** abscess, computed tomography, fluoroscopy, percutaneous drainage, ultrasound

## Abstract

Percutaneous abscess drainage is a safe and effective procedure; however, the techniques used can vary considerably between facilities owing to a lack of standardization. In this report, we aimed to describe the imaging modalities, devices, and techniques implemented in our department to provide useful information on how to perform percutaneous abscess drainage successfully and safely. Our technique of percutaneous abscess drainage includes ultrasound (US)- or computed tomography (CT)-guided abscess puncture, switching to fluoroscopy guidance, contrast medium injection into the abscess cavity, hydrophobic guidewire (GW) insertion, and the advancement of a drainage catheter and metal stiffening cannula without the use of a dilator.

## Introduction

Abscesses can occur at various sites, including the liver, spleen, kidney, peritoneal cavity, pleural cavity, mediastinum, bone, and soft tissue. These infections are clinically important as they represent a persistent focus of infection that requires prompt intervention for effective source control. The effectiveness and safety of percutaneous abscess drainage are widely recognized [1,2]. Although percutaneous drainage is a simple procedure involving puncture and catheter placement, the choices concerning imaging modality, devices, and actual techniques vary across facilities. This report aimed to describe basic techniques and technical tips for percutaneous abscess drainage as employed in our department, rather than providing a comprehensive review of various techniques. We focused primarily on the technical aspects of the procedure.

## Technical report

Procedural settings for percutaneous abscess drainage

Percutaneous abscess drainage can be performed at the bedside, in a fluoroscopy room, angiography room, computed tomography (CT) scan room, or hybrid angiography-CT suite. The following description assumes that the drainage procedure is performed in a hybrid angiography-CT suite; however, the procedure can be performed in a fluoroscopy room when ultrasound (US) is used for puncture.

Imaging modality used for puncture

US or CT

US and CT are most commonly used for puncture and have the features outlined in Table 1 [3,4]. An additional advantage of US over CT is that US-guided puncture can be switched to fluoroscopy within a few seconds, whereas CT-guided puncture might take longer. During this transition, the needle position may change. Based on the as low as reasonably achievable (ALARA) principle, US-guided puncture should be used whenever possible. Upper abdominal organs, such as the liver, spleen, and kidneys, are well-visualized on US. Additionally, they move significantly with respiration. US, which can delineate organs in real time, is suitable for puncturing abscesses in these organs and the surrounding areas that show respiratory movement. However, if the patient has thick subcutaneous fat, abscesses may not be visible on US. In such cases, a CT-guided puncture should be considered. Additionally, the paraspinal region, deep pelvic region, and lungs are often poorly visualized on US, and CT-guided puncture is appropriate.

**Table 1:** Advantages and disadvantages of US and CT CT: computed tomography, US: ultrasound Table credits: Yasuyuki Onishi

Imaging modality	Advantages	Disadvantages
US	Real-time observation of the needle tip and the surrounding structures.	Poor visualization of deep tissues.
	Color Doppler US depiction of blood vessels.	Poor image quality in the presence of air or bone.
	No radiation exposure.	Dependence on the operator’s skill.
CT	Clear distinction among fat, soft tissue, and air.	Difficulty of puncture in the craniocaudal direction.
	Clear description of deep tissues.	Radiation exposure.

US Probes

When US is performed, three types of probes can be used for puncture: convex, microconvex, or linear. Convex and microconvex probes have a lower frequency and better delineate deep lesions, such as a liver abscess or pelvic abscess, whereas linear probes have a higher frequency and better delineate superficial lesions, such as a subcutaneous abscess. If space for the echo beam is limited, a microconvex probe is preferred because of its small contact surface.

Needle Guide in US-Guided Puncture

US-guided puncture can be performed with or without (freehand technique) a needle guide fitting the US probe. When a needle guide is used, the needle is advanced in a predetermined trajectory. This technique is suitable for deep, small abscesses [3]. We routinely use a needle guide in US-guided puncture. However, when the puncture path deviates from the predetermined needle guide trajectory, the freehand technique is used.

CT Technique

When CT is used, either a helical scan with gantry movement or CT fluoroscopy without gantry movement is performed. Furthermore, CT fluoroscopy can be used as either continuous or intermittent CT fluoroscopy, the former being superior in that it provides real-time images; however, it increases radiation exposure. In helical CT and intermittent CT fluoroscopy, the needle is advanced between CT image acquisitions, allowing the operator to minimize radiation exposure by stepping away during scanning. Intermittent CT fluoroscopy shortens the time between CT image acquisitions compared to helical scans because there is no gantry movement, thus reducing procedure time. In CT-guided puncture, staying within the transverse plane is crucial, and puncturing in the craniocaudal direction is challenging. This also applies to CT fluoroscopy, wherein the procedure is performed using three transverse CT images. However, some CT scanners with many detectors can display multiplanar reconstruction images aligned with the long axis of the obliquely punctured needle during CT fluoroscopy, allowing intermittent CT fluoroscopic puncture with a high degree of freedom in the craniocaudal direction [5]. When CT-guided puncture is performed in our department, a helical scan or intermittent CT fluoroscopy is used because of less radiation exposure for both the operator and the patient than continuous CT fluoroscopy.

Puncture method: Seldinger technique

The Seldinger technique is one of the two major types of puncture methods for percutaneous abscess drainage [6]. This technique involves puncturing the abscess with an 18-gauge needle, inserting a 0.035-inch guidewire (GW) through the needle into the abscess cavity, leaving the GW in place, and advancing a drainage catheter over the GW into the abscess; this multi-step method is generally suitable for small, deep abscesses that are difficult to drain [7]. The other major approach is the trocar technique. This technique is simpler and faster, involving the single-step placement of a combined drainage catheter and sharp stylet, making it more suitable for large and superficial abscesses [7]. However, the direct, single-step nature of the trocar technique, where a large-bore catheter is placed immediately, carries a higher risk of inadvertent injury to adjacent organs [8]. Therefore, due to the enhanced safety of the Seldinger technique, we utilize this method for drainage in most cases, as presented in the following section.

Devices for drainage

The needle, GW, and drainage catheter are essential devices for the procedure and are described below.

Puncture Needle

An 18-gauge Chiba needle or an 18- or 19-gauge sheathed needle with a plastic outer sheath and a metal inner stylet can be used as a puncture needle. We occasionally use 18-gauge and 20-cm Chiba needles because their sharpness allows for precise tissue penetration, and the needle tip is more clearly visible under fluoroscopy.

GW

A hydrophobic 0.035-inch GW is appropriate, and we use an 80- or 145-cm Fixed Core Wire Guide (Cook Medical) with a J-shaped tip or Amplatz Extra Stiff GW (Cook Medical) with a J-shaped tip. An excessively long GW is not recommended, as the superfluous length impairs handling characteristics and increases technical difficulty during the procedure. The shaft of the Fixed Core Wire Guide is less rigid than that of Amplatz Extra Stiff GW and is less likely to destroy the abscess wall. Therefore, the Fixed Core Wire Guide application is appropriate when the abscess wall is thin. In contrast, the stiffer shaft of Amplatz Extra Stiff GW is useful when a larger drainage catheter (>12-F) is inserted. The J-shaped tip is less likely to penetrate the abscess wall than the straight or angled tips. We do not use hydrophilic GWs because they are slippery and carry the risk of dislodgement from the abscess cavity during the procedure [7]. Additionally, when manipulating a hydrophilic GW advanced from a metal needle, retraction may cause the coating to peel at the needle bevel or result in wire breakage [9]. Advancing a hydrophilic GW through a Chiba needle is not recommended.

Drainage Catheter

We commonly use an 8.5-F Dawson-Mueller Multipurpose Drainage Catheter (Cook Medical), a pigtail drainage catheter with a locking mechanism at the tip that has a low risk of dislodgement. It has a hydrophilic coating on the distal portion of the shaft for easy insertion and can be inserted without a dilator in most cases [7]. It has three types of accessories: a trocar stylet, a metal stiffening cannula, and a flexible stiffening cannula. Both metal and flexible stiffening cannulas can be inserted into the drainage catheter during placement in the Seldinger technique. We routinely use a metal stiffening cannula because it provides higher rigidity to the drainage catheter, allowing the catheter to be more readily advanced along the GW. We strongly advocate the use of fluoroscopic guidance when the metal stiffening cannula is applied, as the metal stiffening cannula easily penetrates the abscess wall and can damage the adjacent organs. The larger the drainage catheter, the larger the pigtail and the stiffer the shaft of the catheter. For small abscesses or when the viscosity of the contents is low, a 7-F drainage catheter may be selected. If the abscess contents are viscous, a drainage catheter thicker than 8.5-F may be used. Dawson-Mueller catheters above 8.5-F are compatible with 0.038-inch GW, whereas those below 7-F are compatible with 0.035-inch GW. Those above 8.5-F can be placed more easily with a 0.038-inch GW than with a 0.035-inch GW. However, we avoid using the 0.038-inch GW because it is incompatible with the 7-F Dawson-Mueller catheter and prefer the 0.035-inch GW owing to familiarity. Straight-type drainage catheters may be used if the abscess is large and the drainage catheter seems unlikely to dislodge.

Step-by-step description of percutaneous abscess drainage

This section describes the US-guided abscess drainage technique with a needle guide and the CT-guided abscess drainage technique with a helical scan. Fluoroscopy is commonly used for catheter placement following abscess puncture [7], and its application in both techniques is detailed below.

US-Guided Abscess Drainage

**Figure 1:**
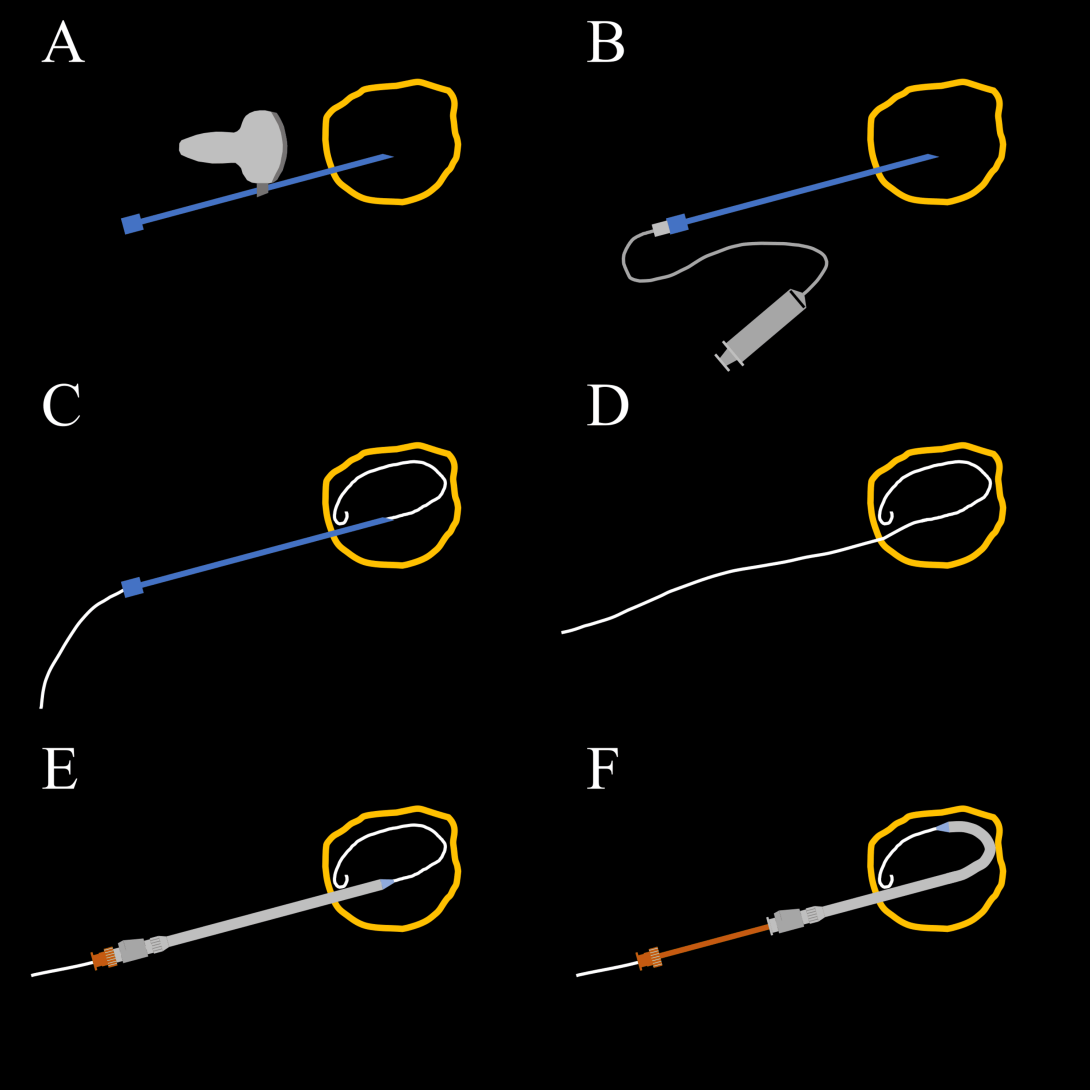
Schematic illustration of US-guided percutaneous abscess drainage procedure (A) The abscess (yellow) is punctured with an 18-gauge Chiba needle using US with a needle guide. (B) The needle is separated from the needle guide, and the inner needle is removed. An extension tube is connected to the Chiba needle. The contrast medium is injected under fluoroscopy. To facilitate subsequent drainage catheter placement, the direction of the X-ray tube should be adjusted such that the needle can be viewed end-on. (C) A 0.035-inch hydrophobic GW is inserted into the abscess cavity under fluoroscopy. It should be advanced sufficiently to create a loop in the abscess cavity. (D) The needle is removed, leaving the GW in place. Therefore, care should be taken to avoid moving the GW. (E) A set of 8.5-F Dawson-Mueller Multipurpose Drainage Catheter and metal stiffening cannula is advanced into the abscess cavity along the GW under fluoroscopy. The direction of advancement of the set should match the direction in which the Chiba needle was punctured. Referring to the fluoroscopic image taken at the time of contrast medium injection into the abscess cavity, the set is advanced to the position of the needle tip. (F) The Dawson-Mueller Multipurpose Drainage Catheter is separated from the stiffening cannula at that position. The drainage catheter is advanced along the GW while the stiffening cannula is kept in place. US: ultrasound; GW: guidewire Image credits: Yasuyuki Onishi

**Figure 2:**
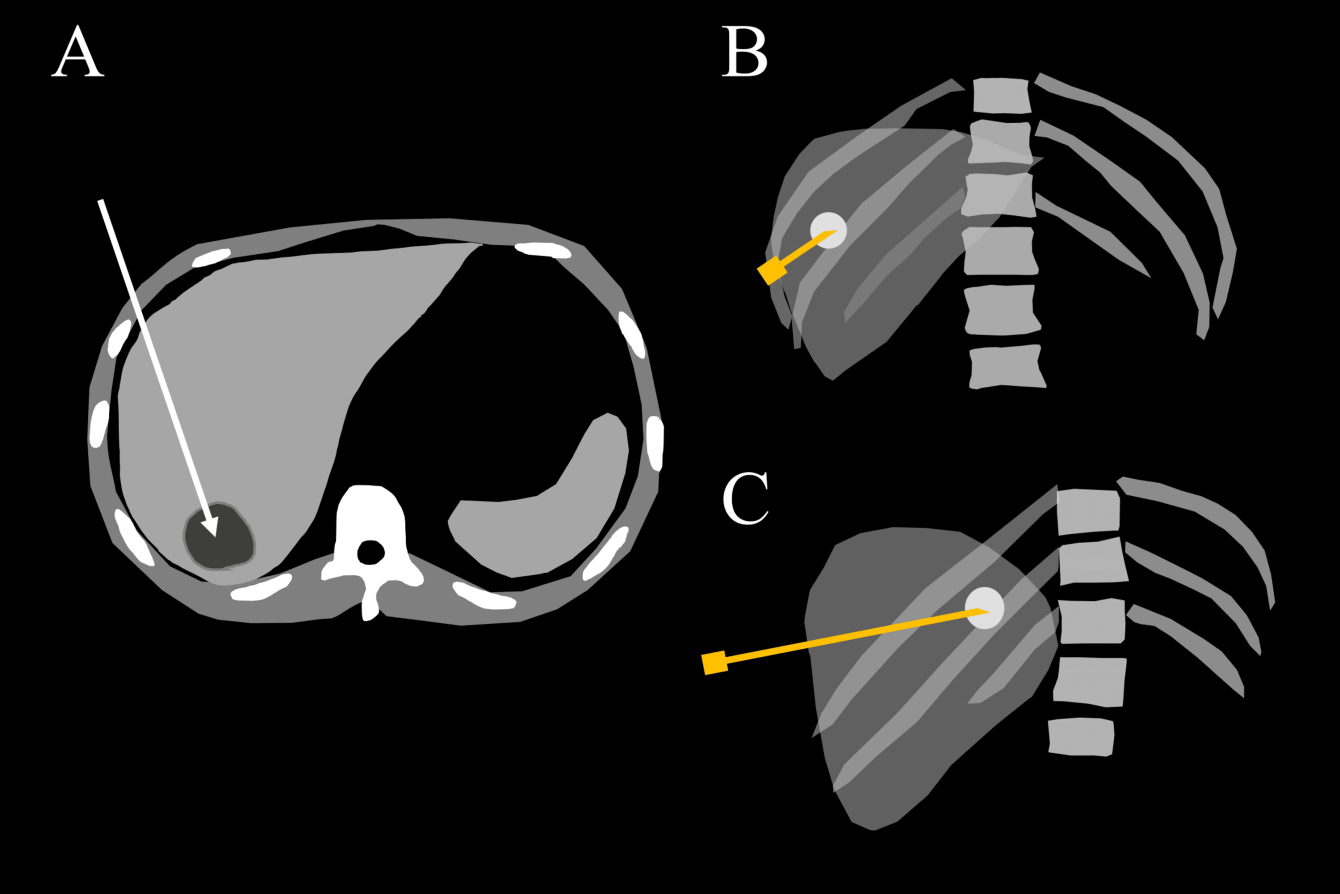
Differences in needle and abscess visualization depending on the direction of the X-ray tube (drainage of a liver abscess is illustrated as an example) (A) Schematic illustration of an axial contrast-enhanced CT image showing an abscess in the posterior lobe of the liver. US-guided percutaneous drainage of the abscess is planned from the right intercostal space. The puncture route is indicated by the white arrow. (B, C) Schematic illustration of the frontal (B) and left anterior oblique (45°) (C) fluoroscopic images obtained after contrast medium injection from the needle. Contrast medium accumulation (white) is observed in the abscess cavity. In the frontal view, the direction of the needle and the direction of the X-ray beam are nearly parallel, making it difficult to determine the relationship between the needle tip and the abscess. In this case, the left anterior oblique view is appropriate for visualizing the relationship between the needle tip and the abscess. It is also easy to adjust the fluoroscopic field of view such that the proximal side of the needle is outside the fluoroscopic field of view, thereby decreasing the radiation exposure to the operator. CT: computed tomography, US: ultrasound Image credits: Yasuyuki Onishi

**Figure 3:**
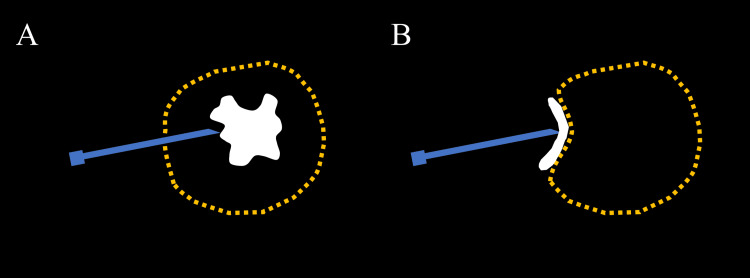
Differences in the spread of injected contrast medium on fluoroscopy (A) When the needle tip is inside the abscess wall (dashed yellow), the contrast medium (white) injected from the needle spreads freely within the abscess cavity. (B) When the needle tip pushes against the abscess wall, the injected contrast medium spreads along the abscess wall. Image credits: Yasuyuki Onishi

1. Determination of body position and route of puncture: The access route is initially planned, and the corresponding patient position is determined by reviewing preceding CT or MRI scans. While the supine position is typically selected for patient comfort, the prone or lateral decubitus positions may be required depending on the target lesion's location and the planned trajectory. It is noteworthy that positions that are heavily tilted, or the lateral decubitus position, are often unstable and carry the risk of the patient's body position changing over time. After appropriate patient positioning, the abscess is assessed by US to determine the puncture route. When puncturing a site with respiratory movement, the optimal puncture route is determined while the patient breathes freely. It is advisable to choose a route that allows abscess puncture at the end of expiration, as the positions of organs are more stable at this time [10]. Furthermore, utilizing color Doppler to confirm the absence of blood vessels in the puncture route is not only safe but is considered good clinical practice.

2. Local anesthesia: After sterile US gel is applied to the skin, a US probe is placed to confirm the puncture path. When the skin surface is even, the saline solution functions as a gel [11]. It can be dripped onto the skin using a syringe or wet gauze. The advantages of saline solution are that it is not as sticky as a gel and is safe even if it enters the body. Local anesthesia is administered under US guidance with a needle guide using a 23-gauge 6- or 7-cm needle in the skin, subcutaneous tissue, and muscle along the puncture route. It is important to fix the US probe on the skin and depict the needle tip on the US. Care should be taken to prevent introducing air through the needle because this will result in poor US images. Needle advancement is performed without breath-holding, and if a site with respiratory movement is to be punctured, the needle should be advanced at the end of expiration. If the liver capsule, peritoneum, or pleura are to be passed, sufficient anesthetic should be injected into these structures because they are pain sensitive [12-14]. During local anesthesia using a needle guide, the needle might not be long enough to administer anesthesia in the planned drainage route to the abscess cavity. In such cases, the needle is detached from the needle guide, and anesthetic administration can be performed using the freehand technique.

3. Skin incision: A small incision is sufficient and can be made by placing a scalpel perpendicular to the skin and applying gentle pressure. A mosquito forceps is used to dissect the subcutaneous tissue. Skin incision size should be adjusted with reference to the drainage catheter size.

4. Puncture: Puncture of the abscess is performed with an 18-gauge 20-cm Chiba needle using a needle guide. During needle advancement, the US probe is fixed to the skin to visualize both the needle tip and the abscess. For stable puncture of a site with respiratory movement, the US probe is fixed to delineate the abscess at the end of expiration, and the needle is advanced only when visualized at this expiratory phase. Occasionally, the abscess wall is hard, and indentation of the wall by the needle occurs. Therefore, advancing the needle tip deep enough until it is surely in the abscess cavity is important. Once the needle reaches the abscess cavity, it is separated from the needle guide. When puncturing an abscess in the abdominal or thoracic cavity, the needle is often held in place by the muscles along the puncture route. Leaving the needle free allows it to move with respiratory movements, keeping the tip in the abscess cavity more easily than holding it with a hand. This method is particularly effective for puncturing organs like the liver, which have significant respiratory movement. Figure 1 presents the post-puncture procedure.

5. Aspiration and contrast medium injection: The inner needle is removed, the abscess content is aspirated, and contrast medium is injected into the abscess cavity. Contrast medium is injected to visualize the abscess cavity and facilitate subsequent GW insertion and catheter placement. To reduce needle movement, a syringe is attached to an extension tube, which is connected to the outer needle for aspiration. The extension tube and needle should be connected loosely enough to avoid needle displacement during attachment or detachment. Additionally, when connecting the tube, care should be taken to prevent air from entering the needle. If contrast is injected without aspirating the abscess contents, the pressure inside the abscess may increase, and bacteria may migrate from the abscess into the blood, causing septic shock [15]. Thus, it is advisable to aspirate a few milliliters of the content before contrast medium injection. If the abscess cavity is small, most of the content can be withdrawn by aspiration, and a sample of the content is commonly submitted for analysis if pathogenic bacteria are unknown. Below, explanations are given for cases in which the content can and cannot be aspirated. 

(a) When the content can be aspirated: After aspirating the contents, a new syringe containing the contrast medium is connected to the extension tube, and the contrast medium is injected under fluoroscopy. As described above, it is recommended to inject a smaller amount of contrast agent than that of the aspirated content. The direction of the X-ray tube should be adjusted so that the needle can be seen in an end-on view (Figure 2). By adjusting the X-ray tube to provide an end-on view of the needle, the relationship between the abscess and needle tip can be better visualized, while also preventing the operator’s hand from entering the fluoroscopic field of view. A fluoroscopic image taken during contrast medium injection should be saved, as this image is useful for subsequent drainage catheter placement.

(b) When the content cannot be aspirated: If the content cannot be aspirated from the needle, there are two possibilities. The needle tip is in the abscess cavity, but the abscess is viscous and cannot be aspirated, or the needle tip has not penetrated the abscess wall and is pushing against it. To distinguish between the two, a small amount of contrast medium is injected under fluoroscopy. If it spreads into the abscess cavity, the needle tip is in the abscess cavity. If it spreads along the abscess wall (Figure 3), the needle tip is pushing against the abscess wall. In the latter, if the needle is advanced slightly and the contrast medium is injected again, it is likely that the contrast medium will enter the abscess cavity. Even when the US clearly shows that the needle tip is in the abscess cavity, injecting the contrast medium is worthwhile because it allows for safe GW and drainage catheter insertion.

6. GW insertion: A 0.035-inch hydrophobic GW with a J-shaped tip is advanced from the needle into the abscess cavity under fluoroscopy. Because breathing and body movements may cause the needle tip to become deeper or shallower, the needle tip position should be adjusted such that the needle tip is in the abscess cavity, referring to the spread of the contrast medium in the abscess cavity. The GW may hit the abscess wall immediately after exiting the needle tip and penetrate it with the GW tip. If the needle is pulled back slightly to separate the distance from the abscess wall and the needle tip, and the GW is advanced again, it often advances into the abscess cavity. The GW should be sufficiently advanced to form a loop in the abscess cavity. The GW comprises a floppy distal part and a stiff shaft. The length of the distal floppy part of each GW is different; therefore, it is necessary to determine the length of the floppy part before the procedure. Once the stiff shaft of the GW is advanced into the abscess cavity, a drainage catheter can be easily placed. 

(a) GW insertion without contrast medium injection: The abovementioned technique of fluoroscopically inserting a GW into the abscess cavity after contrast medium injection is a reliable method for abscess drainage. It is particularly useful for deep and small abscesses. When the abscess is large and located superficially, and the needle tip is clearly observed on US, contrast medium injection is occasionally omitted. In this method, after puncturing the abscess, the needle is not detached from the needle guide. The abscess content is aspirated using an extension tube and syringe. Subsequently, a 0.035-inch hydrophobic GW is inserted through the needle under real-time US observation. After advancing the GW approximately 20 cm or feeling resistance, the needle is detached from the needle guide, and fluoroscopic guidance should be used to check the GW position. This technique is simple, fast, and suitable for large and superficial abscesses.

7. Drainage catheter insertion: The Chiba needle is then removed under fluoroscopic guidance, leaving the GW in place. A metal stiffening cannula is inserted into the 8.5-F Dawson-Mueller catheter. The drainage catheter and stiffening cannula are advanced together along the GW into the abscess cavity. The direction of advancement of the set should match the direction in which the needle was punctured. Furthermore, the GW proximal side is placed in a straight line of the puncture route to straighten the GW, and the GW is gently pulled to facilitate advancement of the drainage catheter and stiffening cannula. Referring to the fluoroscopic image at the time of the contrast medium injection into the abscess cavity, the drainage catheter and stiffening cannula are advanced to the position where the needle tip was present. The drainage catheter is separated from the stiffening cannula at that position. The drainage catheter is then advanced along the GW, while the stiffening cannula is kept in place. Care should be taken not to advance the stiffening cannula too deeply, as it will easily penetrate the abscess wall. Once the catheter is advanced into the abscess cavity, the stiffening cannula and GW are removed. Under fluoroscopy, the thread exiting the proximal side of the catheter is pulled to form a pigtail shape at the tip of the catheter, and the thread is fixed. If the pigtail shape cannot be formed properly, for example, the tip of the pigtail is caught in the abscess wall, then it can be formed by rotating the shaft of the drainage catheter.

8. Aspiration and contrast medium injection: The abscess content is aspirated using the drainage catheter. If the viscosity of the abscess content is too high for aspiration, changing to a larger diameter drainage catheter should be considered. After aspiration of the abscess content, contrast medium is injected through the drainage catheter under fluoroscopy to confirm that the contrast medium spreads throughout the abscess cavity, as seen in the pre-procedure images. Subsequently, the content is aspirated under fluoroscopy to confirm that there is no location in the abscess cavity where the drainage did not work well.

CT-Guided Abscess Drainage (Helical Scan)

1. Determination of body position and route of puncture: The supine position is often selected for the procedure. The puncture route is assumed with reference to the previous CT and MRI. A marker is placed on the skin at the puncture site, and CT is performed with breath-holding at exhalation. The puncture route should be determined in the transverse plane of the abscess. If the CT gantry can be tilted, an oblique puncture of up to 15° can be performed in the transverse plane. If the extent of the abscess is difficult to see on unenhanced CT or if blood vessels running near the puncture route are not depicted on unenhanced CT, CT acquisition after contrast medium injection should be considered [16].

2. Local anesthesia: Local anesthesia is performed with a 23-gauge needle along the determined puncture route. The needle is advanced during breath-holding at exhalation. By emitting a beam from the CT gantry to the transverse plane of the planned puncture and advancing the needle in the beam plane, a puncture can be made in the transverse plane as determined by CT. The angle of the needle in the transverse plane is visually assessed by an operator or an assistant. CT is performed to determine the needle position. The 23-gauge needle is stabilized after approximately 2 cm of advancement; therefore, in most cases, there is no need to grasp the needle during CT acquisition. CT should be taken after the operator moves out of the CT room.

3. Skin incision: A small incision is sufficient and can be made by placing a scalpel perpendicular to the skin and applying gentle pressure. A mosquito forceps is used to dissect the subcutaneous tissue. Skin incision size should be adjusted with reference to the drainage catheter size.

4. Puncture: Abscess puncture is performed using an 18-gauge 20-cm Chiba needle. In some cases, the local anesthesia needle is removed, and the Chiba needle is advanced along the path where the anesthesia needle was inserted. In other cases, the anesthesia needle is left in place, and the Chiba needle is advanced parallel to the anesthesia needle from its vicinity (tandem technique) [17]. The tandem technique is useful when the abscess is small because the angle and position of the first anesthetic needle can be used as a guide for advancing the Chiba needle. The 18-gauge 20-cm Chiba needle is harder to stabilize on the skin than the 23-gauge needle used for anesthesia because the Chiba needle is longer and heavier. It is recommended that the needle be advanced deep enough to penetrate muscles and get stabilized, and that the operator should move out of the room before CT is performed. If the abscess is located in a superficial area, an 18-gauge 60-mm sheathed needle that can be easily stabilized on the skin can be used. The needle is advanced until it reaches the abscess, repeating needle advancement and CT acquisition.

5. Aspiration and contrast medium injection: Similar to US-guided drainage, the abscess content is aspirated using an extension tube and syringe. When the abscess content cannot be aspirated, a small amount of contrast medium is injected through the needle, and CT is performed to confirm that the contrast medium has spread into the abscess.

6. GW insertion and subsequent procedures: These procedures are performed under fluoroscopic guidance, like US-guided abscess drainages.

## Discussion

In this report, we provided a detailed, step-by-step explanation of our technique so that readers can perform the drainage procedure using this approach. We focused on the basic technique of percutaneous abscess drainage, while other percutaneous abscess drainage-related aspects, such as pre-procedure evaluation, complications of the procedure, advanced techniques (changing the patient position, transversal of organs, hydrodissection, etc.), and post-procedure care, are not described in this report [2,4,7,18,19]. Various techniques can be performed for percutaneous abscess drainage that differ from ours. For example, the trocar technique can be implemented [4], a hydrophilic GW can be used for catheter placement [20], or serial tract dilatation by dilators can be performed before drainage catheter placement [15]. We do not consider our method necessarily superior to those used at other facilities. Readers are encouraged to compare our method with those of their own facilities, adopting and applying the aspects of our approach that they find beneficial. Few studies are currently available that will provide similarly detailed explanations of basic drainage techniques. We hope that this report can provide useful hints for improving the techniques applied at other facilities.

## Conclusions

We described in detail the percutaneous abscess drainage procedure, including US- or CT-guided puncture and drainage catheter placement under fluoroscopy, the devices used, and technical tips. Physicians should have knowledge of diagnostic modalities, devices, and techniques for performing safe and accurate percutaneous abscess drainage. Applying the detailed technical insights provided herein will enable clinicians to enhance procedural success rates and improve procedural outcomes.
